# Temperature Effects on the Dielectric Properties and Breakdown Performance of h-BN/Epoxy Composites

**DOI:** 10.3390/ma12244112

**Published:** 2019-12-09

**Authors:** Yongzhe Tang, Peng Zhang, Mingxiao Zhu, Jiacai Li, Yuxia Li, Ziguo Wang, Liangsong Huang

**Affiliations:** 1 Key Laboratory for Robot Intelligent Technology of Shandong Province, Shandong University of Science and Technology, Qingdao 266590, China; tyz1996@163.com (Y.T.); sdustzhangpeng@163.com (P.Z.); yuxiali2004@sdust.edu.cn (Y.L.); wangziguo0017@163.com (Z.W.); 2 College of New Energy, China University of Petroleum (East China), Qingdao 266580, China; zhumx@upc.edu.cn (M.Z.); jiacaili@s.upc.edu.cn (J.L.)

**Keywords:** boron nitride, epoxy, composites, thermal conductivity, dielectric properties, breakdown strength

## Abstract

Epoxy–boron nitride composites are promising insulating materials, and it is highly important to understand their insulating performances at different temperatures with different nano-doping amounts. In this study, we investigated the effects of different mass fractions of epoxy–micron hexagonal boron nitride composites on their thermal conductivity, as well as the effects of temperature and mass fraction on their insulating performances. The results demonstrated that the thermal conductivity of epoxy–micron hexagonal boron nitride composites was superior to that of neat epoxy. The thermal conductivity of epoxy–micron hexagonal boron nitride composites increased with the mass fraction of hexagonal boron nitride, and their dielectric constant and dielectric loss increased with temperature. The dielectric constant of epoxy–micron hexagonal boron nitride composites decreased as the mass fraction of hexagonal boron nitride increased, while their dielectric losses decreased and then increased as the mass fraction of hexagonal boron nitride increased. Due to internal heat accumulation, the alternating current breakdown strength of epoxy–micron hexagonal boron nitride composites increased and then decreased as the mass fraction of hexagonal boron nitride increased. Additionally, as the temperature increased, the composites transitioned from the glassy state to the rubbery or viscous state, and the breakdown strength significantly degraded.

## 1. Introduction

Due to their excellent mechanical and insulating properties, epoxy-based materials have been widely applied in electrical equipment [1,2], but they suffer from poor heat dissipation, resulting in heat accumulation during operation and accelerated aging. As a result, electrical equipment utilizing epoxies have relatively short service lives [3,4]. Due to the rapid development of power systems, electrical equipment is becoming increasingly miniaturized, having large capacity and high dielectric strength, resulting in strict requirements for their thermal conductivity and insulating performance [5,6]. Indeed, enhancing the performances of polymer matrix composites by adding inorganic particles has been a popular research topic [7,8]. Boron nitride (BN) nanosheets have been widely applied as high-voltage insulating materials due to their superior thermal conductivity and insulating performance [9]. The addition of micron-sized hexagonal boron nitride (h-BN) particles into an epoxy resin matrix can significantly enhance the thermal conductivity and electrical properties (e.g., dielectric performance and breakdown strength) of composites at room temperature [10]. However, the operating temperatures of electrical equipment are usually above room temperature, making it important to investigate the effects of temperature micron-sized h-BN on epoxy electrical properties.

Currently, thermal conductivity and electric strength of nanocomposites with high thermal conductivities have attracted great attention. Martin et al. investigated the thermal conductivity of epoxy–micron h-BN composites of different particle diameters. At a BN mass fraction of 20%, the thermal conductivity of composites increased by 400% [11]. Alun et al. investigated the effects of BN nanopowders on the thermal conductivity and insulating performance of epoxy polymers. The results indicated that the thermal conductivities of epoxy-BN nanocomposites were significantly higher than that of neat epoxy. The dielectric and breakdown performances of composites were also improved due to the presence of BN nanoparticles; therefore, epoxy-h-BN composites shall be thoroughly investigated [12]. Yanget al. reported that when h-BN was added to an epoxy resin-impregnated insulation paper, its thermal conductivity increased by 139%. Meanwhile, the breakdown strength remained constant and the inhibition of space charges was observed [13]. Zhanget al. investigated the effects of the addition of boron nitride nanospheres into the epoxy resin matrix on the thermal conductivity and insulating performance of composites. The results demonstrated that the dielectric constant of the composite was larger than that of neat epoxy, while the dielectric loss of the composite was lower than that of neat epoxy, additionally, the thermal conductivity of the composite increased by 765% [14]. Yao et al. demonstrated that the presence of nanoparticles, such as MgO and TiO_2_, regardless of their content, increased the dielectric constant of the composites [15]. Peter et al. reported that the dielectric performances of polymer matrix composites by the addition of metal oxide fillers were lower than expected, while the breakdown strength may degrade [16]. Keet al. investigated the electrical properties of core-shell structured nanocomposites, the results indicated that the internal core-shell structure of the composite significantly increased its dielectric constant, while it negatively affected the dielectric constant and a significant negative effect on dielectric losses of composites [17]. Yanet al. modified BN nanosheets using a dielectric barrier discharge plasma excited by bipolar nanosecond pulse, and the modified BN nanosheets were added into an epoxy resin matrix. The results demonstrated that when the mass fraction of BN nanosheets increased from 10% to 20%, both the thermal conductivity and the breakdown strength increased [18]. Loriane et al. demonstrated that the electric field distortion of the composite could be reduced by micron-sized BN as their dielectric constant is the same as epoxy, although the breakdown strengths of epoxy–micro ninorganic filler composites tend to be lower than that of neat epoxy. BN may also hinder the development of electrical treeingconductive pathways during breakdown. Hence, it was proposed that BN nanoparticles may have positive effects on the breakdown performance of composites via space charge suppression [19]. Overall, BN nanosheets can significantly improve the thermal conductivity and electrical properties of composites at room temperature; however, few studies have reported the effects of h-BN on epoxy performance at different temperatures.

In this study, the dielectric and breakdown characteristics of epoxy–micron h-BN composites were investigated at different temperatures. First, epoxy-h-BN composites with different mass fractions were prepared. Then, the thermal conductivities of composites with different mass fractions were investigated. Finally, the mechanism by which temperature affected the electrical properties (e.g., dielectric performance and breakdown strength) of epoxy-h-BN composites with different mass fractions was studied.

The method of preparation and tests of epoxy–micron h-BN composites are discussed in Section 2. Furthermore, the third section presents the characteristics of the composites and analyzes the testing results. Finally, the summary and the conclusion of the present study are presented in Section 4.

## 2. Materials and Methods

### 2.1. Materials

Bisphenol epoxy (E-51, Eponex 1513) and methyl tetrahydrophthalic anhydride (curing agent) were purchased from Runxiang Chemical Co., Ltd., Changzhou, China. 2,4,6-tri (dimethylamino) phenol (DMP-30) was purchased from Shanghai Aladdin Bio-Chem Technology Co., Ltd., Shanghai, China. BN nanosheets (PW-02) were purchased from Jonyetech Ceramics Co., Ltd., Zibo, China.

### 2.2. Preparation of h-BN/EP Composites

Bisphenol epoxy was heated in an oil bath set to 60 °C until no longer viscous, and an epoxy resin matrix was prepared. The masses of epoxy, curing agent, and accelerator were defined as*m*_1_, *m*_2_, and *m*_3_, respectively, and *m*_1_:*m*_2_:*m*_3_*=* 100:80:1. The mass of added h-BN was *(m*_1_+*m*_2_+*m*_3_*)*×*n*, where *n* is the mass fraction of the h-BN in composite. The h-BN was weighed and added into epoxy, followed by stirring for 30 min. Then, methyl tetrahydrophthalic anhydride was added, followed by stirring and dispersed emulsification for 2 h. Finally, DMP-30 was added, followed by stirring for 30 min. The obtained samples were degassed in a vacuum, poured into a mold, and heated for curing [20].

### 2.3. Performance Tests

#### 2.3.1. SEM Imaging

The microstructures of the h-BN/epoxy composite section were observed by scanning electron microscope (SEM). SEM electron gun emits extremely narrow electron beams scanning composite material, which enables the surface of the composite material to emit secondary electrons, and improves the spatial resolution of the test and reduces the damage to the material. The epoxy-h-BN composite is a nonconductive material. Before the test, a gold sputtering thickness of 6nm should be sputtered on the surface of the composite.

**Figure 1:**
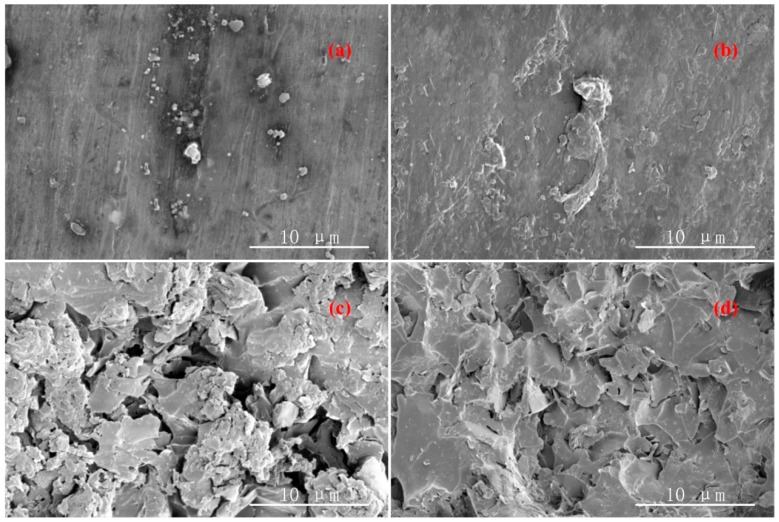
SEM images of epoxy-h-BN composites with mass tractions: (**a**) 1 wt%; (**b**) 3 wt%; (**c**) 5 wt%; (**d**) 7 wt%.

Figure 1 showed the SEM of epoxy-h-BN composites with different mass fractions. As observed, the h-BN particles were coated by epoxy resin matrix and distributed uniformly in epoxy resin matrix without obvious connection when the mass fraction of h-BN packing was low. As the mass fraction of the h-BN filler increased, the distance between h-BN particles decreased and the h-BN particles began to overlap with each other which presented a skeleton structure in the epoxy resin matrix. However, the distribution of h-BN particles in epoxy resin matrix was still relatively uniform and there was no obvious phenomenon of reunion, which might be the specifics area of micron h-BN particles is much lesser than nano h-BN particles and the effects of micron h-BN particles was not obvious.

#### 2.3.2. Thermal Conductivity and Infrared Imaging

The thermal conductivities of composites were measured using amulti-functional thermal conductivity meter (DRE-111; Xiangyi Instrument Co., Ltd., Xiangtan, China) based on the hot disk principle. Using an LED chip as the heat source, surface temperatures of composite samples were measured using a infrared camera (FLIR E6-XT; FLIR Systems, Inc., Wilsonville, OR, USA) and recorded [21].

#### 2.3.3. Dielectric Performance

The dielectric performances of composites with different mass fractions at different temperatures were measured using a high-frequency LCP digital bridge (TH2826; Changzhou Tonghui Electronic Co., Ltd., Changzhou, China). Using the three-electrode method, the protected electrode and shield electrode were placed on samples, and the electrode gap and diameter were measured. Then, positive and negative poles of the digital bridge were connected to ground the bridge. Finally, capacitances and dielectric losses of samples were measured. The dielectric constant of samples $\varepsilon_{r}$ was calculated by:

(1) $$\varepsilon_{r}=\frac{4\times C\times h}{\pi (d_{1}+g)\varepsilon_{0}}$$

where C is the capacitance, h is the thickness, $d_{1}$ is the diameter of the protected electrode, g is the electrode gap, and $\varepsilon_{0}$ is the dielectric constant in a vacuum.

#### 2.3.4. Breakdown Strength

Breakdown strengths of composites with different mass fractions at different temperatures were measured using voltage breakdown equipment (HJC-100KV by Huabotech Co., Ltd., Changchun, China). A ball electrode with a diameter of 25mm was employed, and the voltage was increased at 1 V/s until sample breakdown. The voltage corresponding to the sample breakdown was regarded as the breakdown voltage. The breakdown strength was defined as the ratio of breakdown voltage and average thickness (kV/mm). Samples were immersed in transformer oil whose temperature was varied to measure the breakdown voltages of samples at different temperatures. Ten measurements were performed for each sample.

## 3. Results and Discussion

### 3.1. Thermal Conductivity

**Figure 2:**
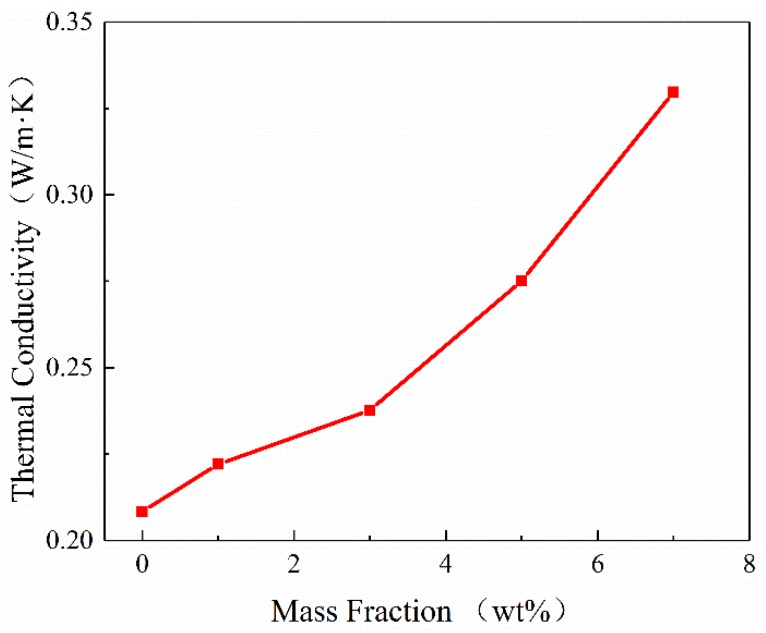
Thermal conductivity values of epoxy-h-BN composites.

Figure 2 shows the measured thermal conductivities of neat epoxy and epoxy-h-BN composites with different mass fractions. The thermal conductivity of composites increased with the massfraction of h-BN. For instance, the thermal conductivity of 7 wt% epoxy-h-BN composite was 58% higher than that of neat epoxy, and the thermal conductivity of all composites containing h-BN was superior to that of neat epoxy. As observed, the thermal conductivity of composites increased with the mass fraction of h-BN.

**Figure 3:**
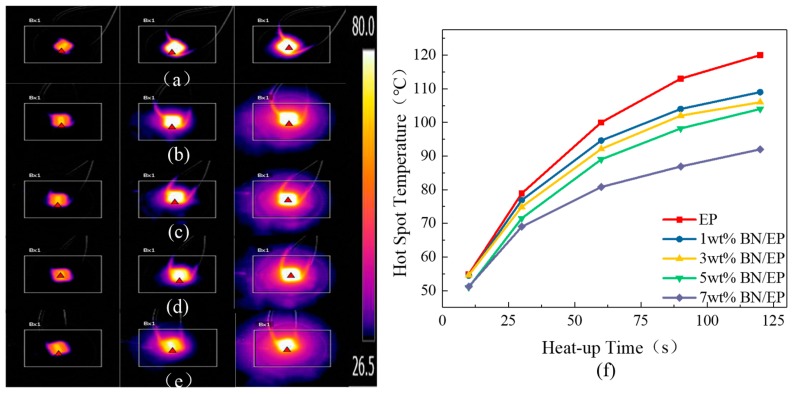
Infrared thermograms of (**a**) neat epoxy, (**b**) 1 wt% h-BN composite, (**c**) 3 wt% h-BN composite, (**d**) 5 wt% h-BN composite, and (**e**) 7 wt% h-BN composite at 10 s, 60 s, and 120 s; (**f**) hot spot temperatures of epoxy-h-BN composites.

Figure 3 shows infrared images and hot spot temperatures of composites. As the mass fraction of h-BN filler increased, the heat distribution ranges of composites at the same moment expanded, the hot spot temperature decreased, and the thermal conductivity increased more rapidly. As a result, the thermal conductivities of composites were significantly enhanced. At low mass fractions of h-BN fillers, h-BN fillers were mixed in the epoxy resin matrix, which increased the thermal conductivity of composites due to the good thermal conductivity of h-BN. However, the quantity of h-BN particles is low, and they tend to form thermally conductive chains. This serial connected heat conduction structure slightly enhanced the thermal conductivity of the composite. As the mass fraction of h-BN increased, the h-BN particles became closer, and more thermally conductive chains were formed, eventually forming a parallel thermally conductive network which significantly enhanced thermal conductivity.

### 3.2. Dielectric Performance

**Figure 4:**
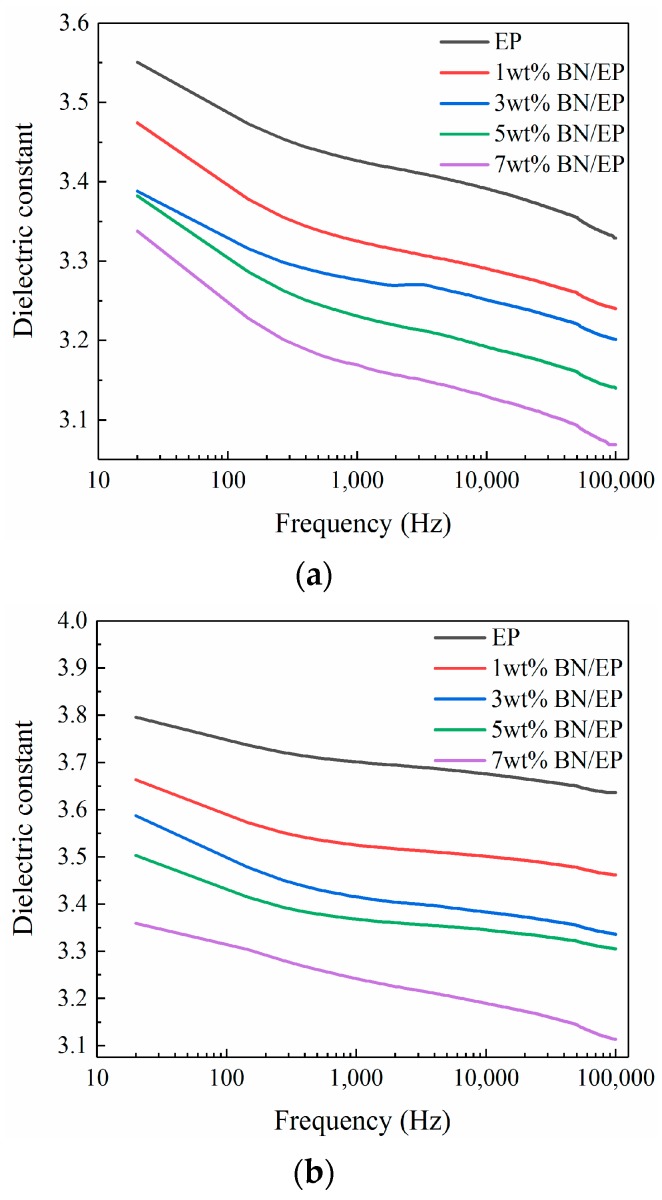

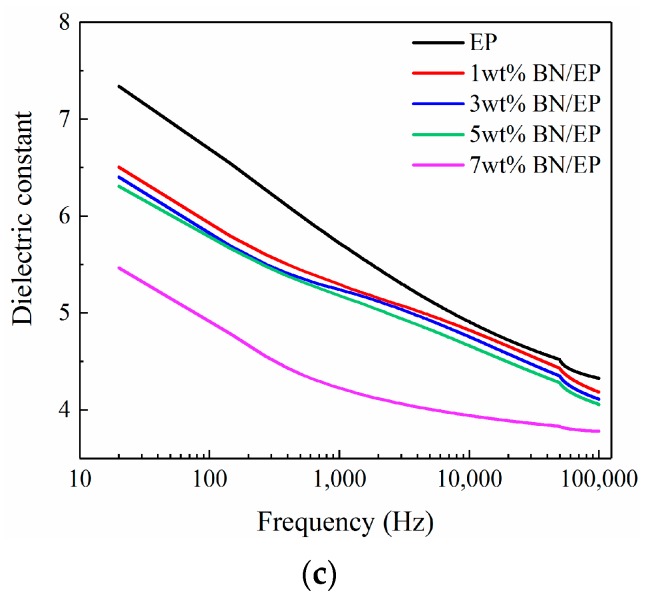
Curves of dielectric constant of epoxy-h-BN composites as a function of frequency at (**a**) 30 °C, (**b**) 90 °C, and (**c**) 150 °C.

**Figure 5:**
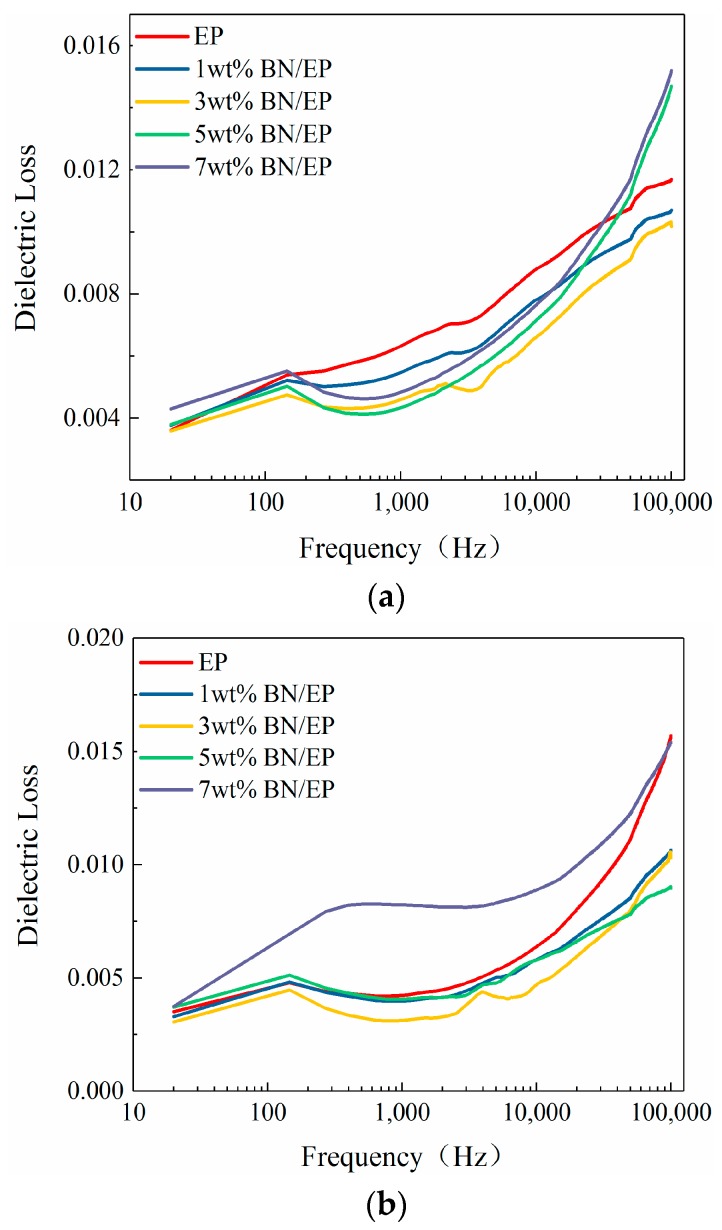

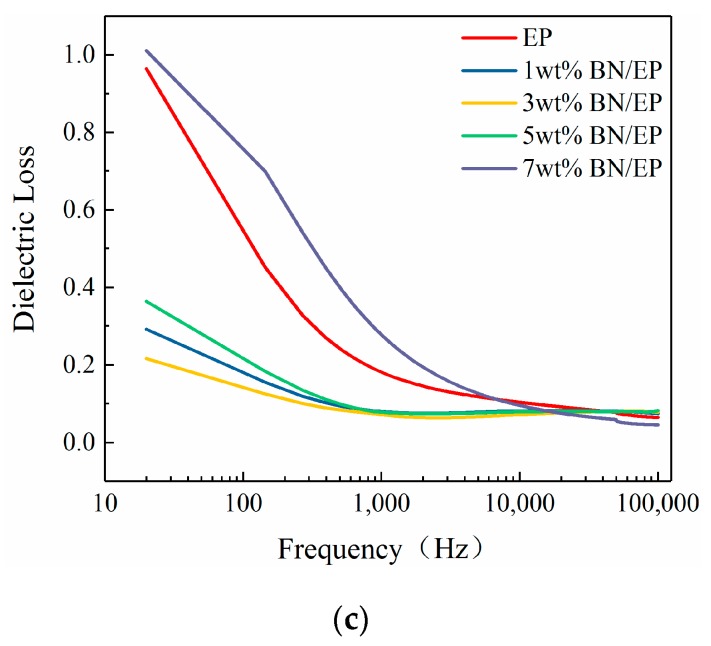
Curves of dielectric losses of epoxy-h-BN composites as a function of frequency at (**a**) 30 °C, (**b**) 90 °C, and (**c**) 150 °C.

Figure 4 and Figure 5 depict the dielectric constant and dielectric loss of neat epoxy and epoxy-h-BN composites with different mass fractions as a function of frequency at 30 °C, 90 °C, and 150 °C.

As shown in Figure 4, the dielectric constants of composites were lower than that of neat epoxy and decreased as the mass fraction of h-BN increased. This may be because the dielectric constant of BN fillers is 3, which is lower than that of the epoxy resin matrix [22,23]. According to the Maxwell–Garnett equation, the dielectric constants of composites can be calculated by [24]:

(2) $$\varepsilon_{r}=\frac{x_{m}*\varepsilon_{m}(\frac{2}{3}+\frac{\varepsilon_{d}}{\varepsilon_{m}})+x_{d}\varepsilon_{d}}{x_{m}(\frac{2}{3}+\frac{\varepsilon_{d}}{\varepsilon_{m}})+x_{d}}$$

where $x_{m}$ is the mass fraction of epoxy, $\varepsilon_{m}$ is the dielectric constant of epoxy, $x_{d}$ is the mass fraction of h-BN fillers, and $\varepsilon_{d}$ is the dielectric constant of h-BN fillers.

According to Equation (2), the dielectric constant of a composite is related to the dielectric constant of the matrix and the mass fraction of fillers. Specifically, the calculated dielectric constant decreased as the mass fraction of fillers increased, which was observed in Figure 4. As shown in Figure 4, the dielectric constants of composites increased with the temperature and the dielectric constant increased more rapidly with the temperature. As the temperature increased, the activity of composite molecules increased, and the hindering effect of the interaction range was reduced. Meanwhile, the activity of polar groups increased, which further enhanced their mobility and transport capacity in electric fields. As a result, the dielectric constant of the composite increased with temperature. Meanwhile, the composites were in a glassy state at low temperatures, and their molecular mobilities were limited. Hence, increasing the temperature had a relatively low effect on the dielectric constant. Once the temperature exceeded the glass transition temperature, the composites were in a rubbery viscous state, and the molecular mobility was enhanced, and increasing the temperature showed a more pronounced effect on the dielectric constant.

As shown in Figure 4, the dielectric constant of composites decreased as the frequency increased because the dielectric constant of a composite is closely related to its polarization effect. At low frequencies, the changing rate of the electric field was low and all polarization processes varied with the electric field. As a result, the dielectric constants of composites tended to be large, and as the frequency increased, the electric field changed more rapidly. However, surface polarization, orientation polarization, and displacement polarization of composites did not vary with the electric field, reducing the dielectric constants of composites.

Figure 5 illustrates the dielectric losses of composites at 30 °C, 90 °C, and 150 °C. As observed, dielectric losses increased with the frequency, and the dielectric loss increased at a faster rate with the frequency at low temperatures (<90 °C), meanwhile, the dielectric losses of composites decreased as the frequency increased, and the dielectric loss drastically degraded as the frequency increased at high temperatures (≥90 °C). This may be attributed to the fact that dielectric losses of composites in an electric field mainly consist of relaxation polarization loss and conductive loss. At low environmental temperatures, the dielectric losses of composites were dominated by relaxation polarization loss of the medium. At high environmental temperatures, the dielectric losses of composites were dominated by conductive loss as kinetic energy, and the mobility of conducting particles in composites increased. At low environmental temperatures, the dielectric losses of composites increased slightly as the frequency increased, although the conductive loss decreased. This was attributed to the fact that the dielectric losses of composites were dominated by relaxation polarization loss. As the frequency increased, the periodic variation of the electric field was consistent with the development time of relaxation polarization. As the relaxation polarization loss increased, the dielectric losses of composites increased drastically. As the environmental temperature increased, conductive loss was inversely proportional to the frequency, and the dielectric losses of composites drastically degraded. As the frequency increased, conductive loss degraded, and relaxation polarization loss was not dominant at high temperatures, causing the dielectric loss to gradually decrease. 

According to dielectric loss curves of composites with different mass fractions in Figure 5, the dielectric losses of composites decreased and then increased as the mass fraction of h-BN increased and reached a minimum at 3 wt%. As the mass fraction of h-BN increased, dielectric losses of composites exceeded those of neat epoxy. In epoxy-h-BN composites, interactions between h-BN particles and epoxy molecules hindered the movements of polar groups in the electric field, thus restraining changes in the dipoles with the electric field during relaxation polarization. In this way, relaxation polarization losses and dielectric losses of composites were reduced. Nevertheless, inter-particle distances decreased at excessively high h-BN mass fractions, and the h-BN readily aggregated [25], increasing the dielectric loss.

### 3.3. Breakdown Strength

In this study, Weibull distributions were employed for data of breakdown strength analysis. The Weibull distribution in this study reflects the breakdown probability of composite samples at a specific field strength. For Alternating Current (AC) breakdown strength, Weibull distributions of the two parameters can be expressed as:

(3) Pf=1−e−(EE0)β

where $P_{f}$ is the accumulated breakdown probability, E is the breakdown field strength, $E_{0}$ is the scale parameter, which refers to the breakdown field strength at breakdown probability of 0.632, andβ is the shape parameter, which is a positive value that reflects the distribution range of breakdown voltage and is inversely proportional to the divergence of breakdown voltages. If the sample size is less than 25, according to the IEC/TC 56 Dependability Standards, $P_{f}$can be expressed as:

(4) $$P_{f}=\frac{i-0.5}{n+0.25}$$

where *i* is the *i*th measured breakdown field strength in ascending order, and n is the quantity of measured breakdown field strengths [26].

**Figure 6:**
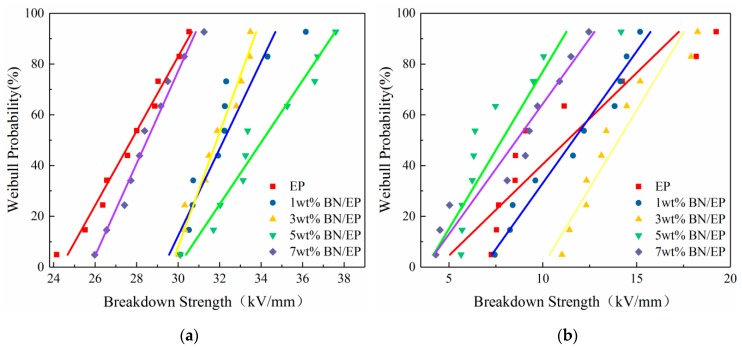
Weibull distribution of breakdown strength of epoxy-h-BN composites at (**a**) 30 °C and (**b**) 150 °C.

Breakdown strengths of neat epoxy and epoxy-h-BN composites with different mass fractions at 30 °C and 150 °C were measured. According to the definition of scale field strength in Equation (3), the breakdown field strength at a breakdown probability of 0.632 was indeed the scale parameter in the Weibull distribution ($E_{0}$). Then, $E_{0}$was substituted into Equation (3), and the shape parameter in the Weibull distribution (β) was calculated based on the measured breakdown field strength (E). Breakdown probabilities were calculated by Equation (4), and the Weibull distributions were depicted (Figure 6). Table 1 illustrates the scale and shape parameters of the Weibull distributions of neat epoxy and epoxy-h-BN composites with different mass fractions at 30 °C and 150 °C.

As shown in Figure 6, the breakdown strengths of composites were higher than that of neat epoxy, and the breakdown strength increased and then decreased as the mass fraction increased. It was observed that the breakdown strengths of composites were affected by their dielectric losses and were maximized at a mass fraction of 3%. The distribution parameters of epoxy-h-BN composites were larger than those of neat epoxy, and the composites displayed a small divergence of breakdown strengths. This may be attributed to the close correlation of composite breakdown in an AC electric field and its internal heat accumulation. For composite breakdown in an AC electric field, the loss power per unit volume *P* can be expressed as [27]:

(5) $$P=E^{2}\omega \varepsilon_{0}\varepsilon_{r}\tan\delta_{0}e^{\alpha (t_{m}-t_{0})}$$

where $\varepsilon_{r}$ is the dielectric constant, $\tan\delta_{0}$ is the tangent fraction of dielectric loss angle at 0 °C, α is the tangent temperature coefficient of dielectric loss angle, $t_{m}$ is the maximum medium temperature, and $t_{0}$ is the environment temperature. In composites, most energy will be converted into heat *Q*:

(6) $$Q=E^{2}h\omega \varepsilon_{0}\varepsilon_{r}\tan\delta_{0}e^{\alpha (t_{m}-t_{0})}$$

As the dielectric constant and dielectric loss of composites increased, heat production increased. When the heat produced exceeded the heat conduction capacity of the medium, heat accumulation was observed, which reduced the breakdown strength. As the mass fraction of h-BN increased, dielectric losses of composites decreased, the heat produced decreased, the thermal conductivity increased, and heat accumulation decreased which enhanced the breakdown strength. The composites displayed minimal dielectric losses and a maximum breakdown strength at an h-BN mass fraction of 3%. As the mass fraction increased further, aggregation was observed, resulting in an increased dielectric loss and heat generation, causing the breakdown strength to decrease. However, due to their enhanced thermal conductivity, the breakdown strengths of composites with high mass fractions were larger than that of neat epoxy.

As shown in Figure 6, the breakdown strengths of composites degraded drastically as the temperature increased. The maximum breakdown strength at 150 °C degraded by 58% compared with that at 30 °C. Once the temperature reached the glass transition temperature, the breakdown strength increased more rapidly. As shown in Table 1, as the temperature increased, the Weibull distribution parameters of composites decreased, the divergence of breakdown strength increased, and the high-temperature stability of composites degraded. This may be attributed to the fact that if the temperature was below the glass transition temperature of the composite, it was in a glassy state, and its molecular structure was stable. In other words, its molecular structure had low mobility, and the breakdown performance remained constant. The decreasing rate of breakdown strength was low. When the temperature exceeded the glass transition temperature of the composite, the composite entered a rubbery state, and the molecular structure experienced significant changes under the influence of the external electric field. In this case, its performance was significantly affected by the temperature, the dielectric loss increased drastically, and its breakdown strength rapidly degraded.

On the other hand, the interaction range of h-BN particles and the epoxy resin matrix enhanced the compactness of the overall composite structure. Hence, the breakdown strengths of composites with low mass fractions of h-BN increased; however, as the mass fraction increased, a path formed in the interaction range of h-BN particles and the epoxy resin matrix which facilitated the development of a breakdown path that reduced the breakdown strength of the composite. As the temperature increased, molecular movements accelerated, and the inhibition effect of the interaction range degraded, resulting in reduced breakdown strengths of composites [28].

## 4. Conclusions

In this study, epoxy–micron h-BN composites with different mass fractions were prepared and their thermal conductivities were investigated. Indeed, this study focused on the dielectric performances and AC breakdown performances of epoxy–micron h-BN composites at different temperatures, as well as their working mechanisms.

The conductivities of all composites were higher than that of neat epoxy. The thermal conductivities of composites increased with the mass fraction of h-BN, and the increase became more rapid at higher h-BN mass fractions. This may be attributed to the thermally conductive chains and networks of h-BN in composites.

As the temperature increased, molecular mobility was enhanced, and both dielectric constants and dielectric losses of composites increased. The dielectric constants of composites also increased as the frequency decreased. The dielectric losses of composites increased at low temperatures and decreased at high temperatures as the frequency increased. This may be attributed to the transition of the dominant component of dielectric loss from relaxation polarization loss to conductive loss at higher temperatures. As the mass fraction of h-BN increased, the dielectric constant decreased, while the dielectric loss decreased and then increased. The minimum dielectric loss was observed at an h-BN mass fraction of 3%.

As the mass fraction increased, the breakdown strength of composites increased and then decreased. At an h-BN mass fraction of 3%, the breakdown strength reached a maximum. Meanwhile, the breakdown strength and dielectric loss showed the same trend, possibly due to heat accumulation during breakdown. At the mass fractions used in this study, the breakdown strengths of all composites were larger than that of neat epoxy, indicating that molecular interactions in composites positively affected their breakdown strengths. When the temperature increased above the glass transition temperature, composites were more affected by temperature, and their breakdown strengths decreased drastically. The maximum breakdown strength was 58% lower compared with that at 30 °C.

## Figures and Tables

**Table 1:** Weibull distribution scale parameters and shape parameters of neat epoxy and epoxy-h-BN composites with different mass fractions at 30 °C and 150 °C.

Mass Fraction	Scale Parameter *E*_0_ (30 °C/150 °C)		Shape Parameter β (30 °C/150 °C)	
Neat epoxy	28.8807	11.1500	10.4620	2.2280
1 wt%	32.2537	13.8325	18.0430	2.3940
3 wt%	32.8070	14.4751	18.3060	5.4410
5 wt%	35.2565	7.48244	14.0080	4.8110
7 wt%	29.1689	9.72113	17.0990	4.7950
